# Synaptic Loss, ER Stress and Neuro-Inflammation Emerge Late in the Lateral Temporal Cortex and Associate with Progressive Tau Pathology in Alzheimer’s Disease

**DOI:** 10.1007/s12035-020-01950-1

**Published:** 2020-06-08

**Authors:** Heather Buchanan, Murray Mackay, Kerri Palmer, Karolína Tothová, Miroslava Katsur, Bettina Platt, David J. Koss

**Affiliations:** 1 School of Medicine, Medical Sciences and Nutrition, University of Aberdeen, Foresterhill, Aberdeen, AB25 2ZD UK; 2 Institute of Neuroscience, Campus for Ageing and Vitality, Newcastle University, Newcastle upon Tyne, NE4 5PL UK

**Keywords:** Alzheimer’s disease, Tau, Amyloid-β, Synapse, Unfolded protein response, Neuro-inflammation

## Abstract

The complex multifactorial nature of AD pathogenesis has been highlighted by evidence implicating additional neurodegenerative mechanisms, beyond that of amyloid-β (Aβ) and tau. To provide insight into cause and effect, we here investigated the temporal profile and associations of pathological changes in synaptic, endoplasmic reticulum (ER) stress and neuro-inflammatory markers. Quantifications were established via immunoblot and immunohistochemistry protocols in post-mortem lateral temporal cortex (*n* = 46). All measures were assessed according to diagnosis (non-AD vs. AD), neuropathological severity (low (Braak ≤ 2) vs. moderate (3–4) vs. severe (≥ 5)) and individual Braak stage, and were correlated with Aβ and tau pathology and cognitive scores. Postsynaptic PSD-95, but not presynaptic synaptophysin, was decreased in AD cases and demonstrated a progressive decline across disease severity and Braak stage, yet not with cognitive scores. Of all investigated ER stress markers, only phospho-protein kinase RNA-like ER kinase (p-PERK) correlated with Braak stage and was increased in diagnosed AD cases. A similar relationship was observed for the astrocytic glial fibrillary acidic protein (GFAP); however, the associated aquaporin 4 and microglial Iba1 remained unchanged. Pathological alterations in these markers preferentially correlated with measures of tau over those related to Aβ. Notably, GFAP also correlated strongly with Aβ markers and with all assessments of cognition. Lateral temporal cortex-associated synaptic, ER stress and neuro-inflammatory pathologies are here determined as late occurrences in AD progression, largely associated with tau pathology. Moreover, GFAP emerged as the most robust indicator of disease progression, tau/Aβ pathology, and cognitive impairment.

**Electronic supplementary material:**

The online version of this article (10.1007/s12035-020-01950-1) contains supplementary material, which is available to authorized users.

## Introduction

Alzheimer’s disease (AD), the most common cause of dementia, is characterised by the accumulation of extracellular amyloid-β (Aβ) containing plaques and intracellular neurofibrillary tangles (NFTs) composed of hyper-phosphorylated tau. These hallmark features have largely been the focus of AD research; for decades, the ‘amyloid cascade hypothesis’ [1] remained the foremost pathogenic concept in the field and has since been revised to incorporate a role for earlier occurring soluble species, which appear to hold more disease relevance [2–4].

Attempts at effective pharmacological interventions have thus far focussed on addressing the development of Aβ, and more recently tau, pathology. However, after consistent failures in clinical trials, we are still without a disease-modifying agent [5]. This has called into question the validity of current hypotheses and our conceptual understanding of disease aetiology. Certainly, the sequential spread of tau and Aβ across brain regions exemplifies inherent differences of these key pathological markers. For instance, cortical tau pathology starts in the entorhinal cortex (EC) and then spreads to the hippocampus and lateral temporal lobes before reaching the remaining areas of the neocortex [6]. In contrast, Aβ plaques are first observed in the neocortex and reach the hippocampus and EC in later stages [7], potentially depositing first in the inferior temporal gyrus or orbitofrontal cortices [8, 9]. Furthermore, the separation between Aβ and tau is evident in their correlative strength with disease status and/or cognitive decline. Despite the genetic association of Aβ with AD, it is tau pathology, genetically linked to frontotemporal lobar degeneration (FTLD), which is the more robust indicator of disease progression and cognitive decline [2, 3, 10]. Alongside the apparent ‘hallmark’ pathologies, several additional tissue alterations are also apparent and assumed to contribute to either onset or progression of degenerative processes [11]. Indeed, ageing itself and also numerous homeostatic pathways have been linked to neurodegeneration, including those associated with oxidative stress, inflammation, vascular and metabolic dysfunction [12].

Here, we focussed on putative disease-relevant pathways associated with synaptic pathology, neuro-inflammation and endoplasmic reticulum (ER) stress (see [13–15] for review). Synaptic loss, thought to occur early in the disease process, may hold stronger functional relevance than the hallmark pathologies and may precede neuronal degeneration [16, 17]. Indeed, a synaptic loss of 20–40% in the hippocampal region has been reported in early AD [18, 19].

Similar to the loss of synapses, evidence for ER stress in the form of the unfolded protein response (UPR) as signalled by activation of protein kinase RNA-like ER kinase (PERK), inositol-requiring enzyme 1α (IRE1α) and activating transcription factor 6 (ATF6), has also been reported in the early stages of AD [20–22] and associated animal models [23–25]. Early activation of the UPR appears most prominent in the hippocampus [26, 27]. Downstream from PERK, the phosphorylation of eukaryotic initiation factor 2α (p-eIF2α) is considered a major output of the UPR associated with the inhibition of protein translation leading to impaired synaptic plasticity, learning and memory and, thus, may connect the UPR with synaptic deficits [23, 28, 29].

Associated with both synaptic loss [30] and ER stress [31], aberrant central nervous system inflammation has also been readily proposed as a contributing factor to AD. Neuro-inflammation is a well-characterised feature of neurodegenerative events; its relevance has been highlighted through genetic mutations associated with receptors of the innate immune system and AD risk [32, 33]. Additionally, numerous studies report alterations in astrocytic markers such as GFAP [34, 35] and aquaporin 4 (AQP4) [36], as well as microglial markers [15] in AD states.

Given the complex nature and likely interdependence of the above-mentioned pathways, it is vital to understand how they are initially activated and spread throughout the brain. Equally, it is critical to determine primary vs. secondary events via an analysis of associations with the established spatio-temporal progression of principal hallmarks. Synaptic loss, the UPR and neuro-inflammation have each been implicated as early occurring cascades, evident in key brain regions foremost affected at the initial stages of the disease (for example, see [26, 37, 38]). Conceptually, the activation of such cascades in regions affected at later stages, prior to the emergence of regional Aβ and tau pathology, would support a causative role in the spread of the disease, whilst later stage detection following robust pathology would support a more reactive downstream role for these stressors.

Here, we utilise a post-mortem tissue cohort of non-diseased and AD cases, previously characterised for a variety of tau and Aβ species [2, 3], to contextualise the activation of the UPR and neuro-inflammation as well as associations with synaptic loss within the lateral temporal cortex. Indicative markers were determined according to diagnosis, pathological severity and neuropathological staging. Additional correlative analyses were performed with established Aβ and tau markers as well as cognitive assessment scores.

## Materials and Methods

### Post-mortem Human Brain Samples

Human temporal cortex samples (middle temporal gyrus, Brodmann’s area 21) were obtained from the Brains for Dementia (BDR) consortium and sourced from the MRC London Neurodegenerative Diseases Brain Bank, the Thomas Willis Oxford Brain Collection, the Manchester Brain Bank, the Newcastle Brain Tissue Resource and the South West Dementia Brain Bank. Prior to patient death, informed consent was attained. All procedures were approved by the UK Medical Research Council.

Samples were received as either 500-mg frozen blocks (*n* = 46) or 5-μm-thick paraffin-embedded sections (*n* = 28) for western blot or immunohistochemical analysis, respectively. Cases were deemed as non-AD or AD, determined by clinical and neuropathological assessment, as per medical history and neuropathological assessments received by the BDR. Corresponding biographical data including sex, age at death, post-mortem interval (PMI), cortical pH, Braak staging, CERAD neuritic plaque score, National Institute on Aging and Alzheimer’s Association (NIA-AA) guidelines reporting none, low, intermediate (Inter) or high neuropathic changes related to AD and cognitive scores (Mini Mental State Examination (MMSE); Clinical Dementia Rating (CDR) global, memory and sum of box) were also supplied where detailed. No significant differences in PMI or age were detected across analytical groups (see Table 1 for details and Table S1 for additional information relating to Thal phase, the assessment of primary age-related tauopathy (PART; as per guidelines in Crary et al. [39]) and apolipoprotein Ɛ4 allele status of cases used).

**Table 1:** Biographical information of the study cohort. Number of cases, CERAD score, NIA-AA classification (none, low, intermediate (Inter) and high degree of AD-related neuropathological change), sex (% male), age at death, post-mortem interval (PMI) and cortical pH are indicated, stratified according to diagnosis, neuropathological severity and individual Braak stage. Mean values presented alongside ± SEM

	No. cases	CERAD	NIAA	Male (%)	Age range (years)	Mean age (years)	PMI range (h)	Mean PMI (h)	pH range	Mean pH
Diagnosis (Braak stage)										
Non-AD (0–3)	27	C0-C2	None–Inter	44.4	74–103	86 ± 1.4	11–110	44.9 ± 5.2	5.4–6.9	6.3 ± 0.1
AD (4–6)	19	C2-C3	Inter–High	60	71–90	83.4 ± 1.2	20–87	46.6 ± 4.9	6–6.9	6.3 ± 0.1
Severity (Braak stage)										
Low (0–2)	18	C0-C1	None–Low	66.7	74–103	85.3 ± 1.9	11–92	40.7 ± 6.2	6–6.7	6.3 ± 0.1
Moderate (3–4)	14	C0-C3	None–Inter	28.6	77–95	85.9 ± 1.5	13.5–101	51.5 ± 7	5.4–6.9	6.2 ± 0.2
Severe (5–6)	14	C1-C3	Inter–High	71.4	71–90	83.6 ± 1.5	20–78	46 ± 5.7	6.1–6.9	6.4 ± 0.1
Braak stage										
0	3	C0	None	100	74–78	76.7 ± 1.3	11–56	30 ± 13.5	6–6.1	6.1 ± 0.1
2	15	C0-C1	None–Low	40	74–103	87 ± 2	12–92	42.8 ± 7	6–6.7	6.3 ± 0.1
3	9	C0-C2	None–Inter	33.3	78–95	87.6 ± 1.7	13.5–101	53.4 ± 9.5	5.4–6.9	6.2 ± 0.3
4	5	C2-C3	Inter	40	77–88	82.8 ± 2.2	26–87	48.2 ± 10.6	6.0–6.8	6.2 ± 0.2
5	6	C1-C3	Inter–High	83.3	82–88	83.8 ± 1.2	22–78	50.5 ± 10.1	6.1–6.6	6.3 ± 0.1
6	8	C3	Inter—High	50	71–90	83.5 ± 2.4	20–69	42.6 ± 6.9	6.3–6.9	6.5 ± 0.1

### Brain Lysate Preparation

Frozen tissue (100 mg) was manually homogenised in ~ 1:10 (w/v) Igepal/NP-40 (Sigma, Dorset, UK)-based lysis buffer (in mM: 20 HEPES, 150 NaCl, 1% Igepal/NP-40, 0.1 EDTA, pH = 7.6) including protease and phosphatase inhibitors (cOmplete mini and PhosStop, Roche Life Science, Burgess Hill, UK). Homogenates were centrifuged (13,000*g*, 4 °C, 20 min) and supernatants containing soluble material were stored at − 80 °C.

### Western Blotting

Generated soluble lysates from each case were probed for markers of synaptic integrity (postsynaptic density 95 (PSD-95), Abcam—cat. # ab18258 and synaptophysin, Abcam—cat. # ab32127), ER stress (binding immunoglobulin protein, BiP), Abclonal—cat. # A0241; p-PERK, Cell Signalling—cat. # 3179s; PERK, Cell Signalling—cat. # 3192s; p-eIF2α, Cell Signalling—cat. # 9721s; eIF2α, Cell Signalling—cat. # 9722s; p-IRE1α, ThermoFisher—cat. # PA1-16927 and IRE1α, Cell Signalling—cat. # 3294s) and neuro-inflammation (GFAP, Sigma—cat. # G3893 and Iba1 cat. # 016-20001) (see Table S2 for further details).

Samples were subject to standard western blot protocols as described previously [3]. Protein concentration was determined through bicinchoninic acid assay (BCA, Sigma) and samples adjusted to the desired concentration in lithium dodecyl sulphate (LDS, Thermo Fisher, Paisley, UK) and 15 mM dithiothreitol (DTT, Sigma). Samples were heated (70 °C, 10 min) and separated on 4–12% Bis-Tris gels (ThermoFisher) in either MOPS or MES SDS running buffer (ThermoFisher). Proteins were transferred onto 0.2 μm or 0.45 μm nitrocellulose membranes via standard wet transfer conditions. Membranes were washed in 0.05% Tween-20 (Sigma) containing Tris-buffered saline (TBST; in mM: 50 Trizma base, 150 NaCl, pH = 7.6), before blocking for 1 h at room temperature in TBST containing 5% milk powder. Primary antibodies were incubated overnight (4 °C) in TBST containing 5% bovine serum albumin (BSA).

Appropriate secondary antibodies were applied for 1 h at room temperature (goat anti-rabbit/goat anti-mouse, IgG, HRP conjugated; Merck Millipore (1:5000)) prior to visualisation using enhanced chemiluminescence (1.25 mM luminol, 30 μM coumaric acid, 0.015% H_2_O_2_). Membranes were washed in TBST (3 × 5 min) between each stage of the protocol. Immunoreactivity was captured using a Vilber-Fusion-SL camera (Vilber, Eberhardzell, Germany) at 16-bit for analysis and 8-bit for illustration. Membranes were then re-probed for total protein in the case of phospho-markers, as above, or stained for total protein using Coomassie total protein stain (see Fig. S1 for example of Coomassie-stained membranes) as previously described [2, 3].

Supportive native state dot blots (as per [2, 3]) for AQP4 were conducted due to the incompatibility of the antibody with western blotting protocols.

### Immunohistochemistry

Fixed brain sections were de-waxed in xylene and rehydrated in ethanol followed by a 20-min antigen retrieval treatment in boiling citric acid solution (10 mM citric acid, 0.05% Tween-20, pH = 6). After blocking for 1 h at room temperature in phosphate-buffered saline (PBS; 0.01 M) containing 1.5% normal goat serum, 1% milk powder, 2% BSA and 1% triton, primary antibodies against GFAP (Alexa 488 conjugated, 1:500; Novus Biologicals, Abingdon, UK), Iba1 (1:200, Wako) and AQP4 (1:100, Merck Millipore) were incubated overnight at 4 °C. Additional sections with no primary antibody served as secondary antibody controls. Secondary antibodies were applied (goat anti-rabbit Alexa 594, 1:500, ThermoFisher) for 1 h at room temperature in PBS containing 2% BSA before mounting (Prolong® Diamond Antifade Mountant with DAPI, ThermoFisher). Where appropriate, sections were washed in PBS. Sections were viewed and captured (15 images per section; randomised fields independent of grey or white matter) using an Axioskop 2 plus microscope (Zeiss, Cambridge, UK) and Axiovision software. From the 28 cases for which histology sections were available, no fewer than a total of 24 cases (1 section per case) were assessed for each marker (for specifics of each marker, see relevant figure legends).

### Quantification and Cohort Stratification

Quantification was conducted using ImageJ (ver. 1.47, NIH, USA) software. For western blot analysis, immunoreactivity was quantified according to area under the curve (AUC) measurements and adjusted to respective total protein markers (for phospho-markers) or total protein Coomassie stain. Adjusted values were subsequently normalised to control groups within blots (according to classifications below) before all data for each marker were pooled.

For immunohistological analysis, captured images were quantified according to area stained (15 images per section were averaged to give overall % area stained, and then data was pooled as below). Equal exposure, background and threshold settings were implemented. For all markers, cases within the cohort were processed in batches, with comparisons between runs made to ensure reproducibility. Thus, the quantification of a marker represents pooled measures across multiple blotting/staining runs.

For all data, values were expressed relative to appropriate controls and analysed according to the following three classifications [2, 3]:

1. Clinical *diagnosis* of AD, confirmed post-mortem (non-AD = Braak 0–3; AD = Braak 4–6)
2. Neuropathological phospho-tau *severity*, grouped as low (Braak 0–2), moderate (Braak 3–4) or severe (Braak 5–6)
3. Individual *Braak stages* (normalised to Braak stage 2)

Additional analysis reporting the impact of overall AD relevant neuropathological change was conducted following the cohort stratification according to the National Institute of Aging-Alzheimer’s Association guidelines (NIA-AA) as determined by Braak neurofibrillary tangle staging, Thal phases and CERAD scores [40]. Furthermore, the potential association of each marker with APOE-related risk (as determined by ε4ε4 = 2; ε3ε4 = 1; ε3ε3 = 0; ε2ε4 = 0; ε2ε3 = − 1; ε2ε2 = − 2 scoring) was also conducted.

### Statistical Analysis

Statistical analysis was conducted using Graphpad Prism 5 software. Normal distribution was probed via a Shapiro-Wilk test. Comparisons of two data sets were conducted using Student’s two-tailed *t* test, with Welsh correction if appropriate, or Mann-Whitney test for non-parametric data. A one-way analysis of variance (ANOVA) or Kruskal-Wallis test was conducted for multiple group analysis. Where statistical significance was indicated, Bonferroni or Dunn’s post hoc tests were implemented. Spearman’s rank correlation coefficient (*r*) was used to determine correlations and probable error (P.E.) of coefficient was calculated for each significant correlation. For all data, the level of significance was set as *p* < 0.05.

## Results

### Synaptic Pathology

**Fig. 1:**
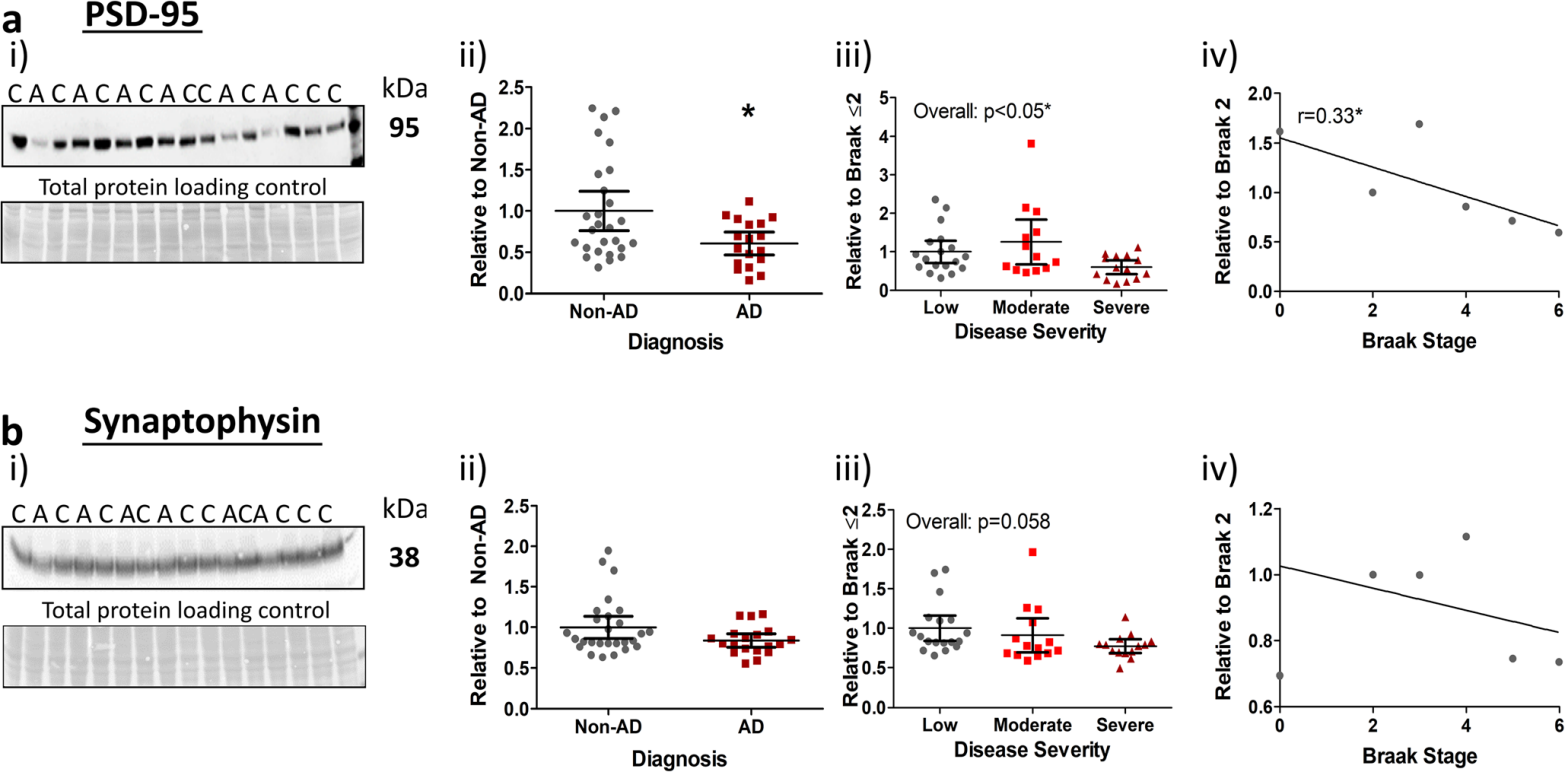
Postsynaptic markers decrease with AD progression. i) Representative images of western blots probed for **a** PSD-95 and **b** synaptophysin with molecular weights and diagnosis of non-AD (controls, (C)) and AD (A) indicated. An area of each blot stained for total protein loading is additionally shown as means of a loading control. Quantified markers were stratified according to ii) diagnosis, iii) disease severity (low, Braak 0–2; moderate, Braak 3–4; severe, Braak 5–6) and iv) individual Braak stage for Spearman’s rank correlation (*r*) analysis. Significant post hoc inter-group differences are indicated (*). Data for PSD-95 (*n* = 45) and synaptophysin (*n* = 46) are provided as scatter plots with means with 95% confidence intervals, *p < 0.05

Synaptic loss and its associations with AD pathology and progression was first determined via immunoblotting of the postsynaptic marker, PSD-95 and the presynaptic marker, synaptophysin. For clinically and neuropathologically diagnosed AD cases, reduced levels of PSD-95 were confirmed (Fig. 1a(i, ii), *p* < 0.05). PSD-95 levels also declined with disease severity (Fig. 1a(iii), *p* < 0.05), without significant change between the 3 severity classifications, yet in agreement with Braak stages (Fig. 1a(iv), *p* < 0.05, *r* = 0.33, P.E. ± 0.14). Together, our results confirmed the progressive loss of this postsynaptic marker for all group stratifications.

In contrast, the presynaptic marker synaptophysin was unchanged across diagnosis (Fig. 1b(i, ii)) and individual Braak stage (Fig. 1b(iv)), although a strong trend emerged when analysed across disease severity (Fig. 1b(iii), *p* = 0.058). This suggests that the presynaptic compartment is overall less affected in AD, and only marginally so in comparison to its postsynaptic counterpart. Interestingly, however, when samples with additional pathologies not directly related to AD were excluded (*n* = 5; see Table S1 for cases excluded), a significant reduction in synaptophysin levels was detected (non-AD vs. AD (*p* < 0.05); Braak stage (*p* < 0.05, *r* = − 0.36, P.E. = ± 0.15); see Fig. S2), indicating a possible impact of secondary pathologies on the presynaptic compartment. For all other markers analysed, exclusion of additional pathologies yielded no marked changes in statistical outcomes.

Surprisingly, correlative analysis between synaptic pathology, cognitive scores and other AD-related pathology markers [2, 3] revealed that neither synaptic marker correlated with CERAD (neuritic plaque) or cognitive scores from multiple assessments (*p* > 0.05 for all). Furthermore, neither marker correlated with Aβ pathology, but both demonstrated some degree of correlation with tau, specifically phospho-tau, measures (Table 2).

**Table 2:** Correlations between synaptic markers with β-amyloid and tau pathology. Spearman’s rank correlations (*r*) between synaptic, amyloid-β (Aβ) and tau markers are reported. Negative correlations are indicated by arrows. *p < 0.05, **p < 0.01, ^$^trend. *N.S.* not significant

Marker	Soluble Aβ	Fibrillar Aβ	Oligomeric tau	Phosphorylated tau			Total tau
	MOAB	OC	TOC-1	PHF-1	AT-8	CP-13	HT-7
PSD-95	N.S.	N.S.	N.S.	*r = 0.33 ± 0.14 (↓)	*r = 0.3 ± 0.14 (↓)	N.S.	N.S.
Synaptophysin	N.S.	N.S.	N.S.	N.S.	^$^*p* = 0.07 (↓)	N.S.	N.S.

### UPR Pathology

**Fig. 2:**
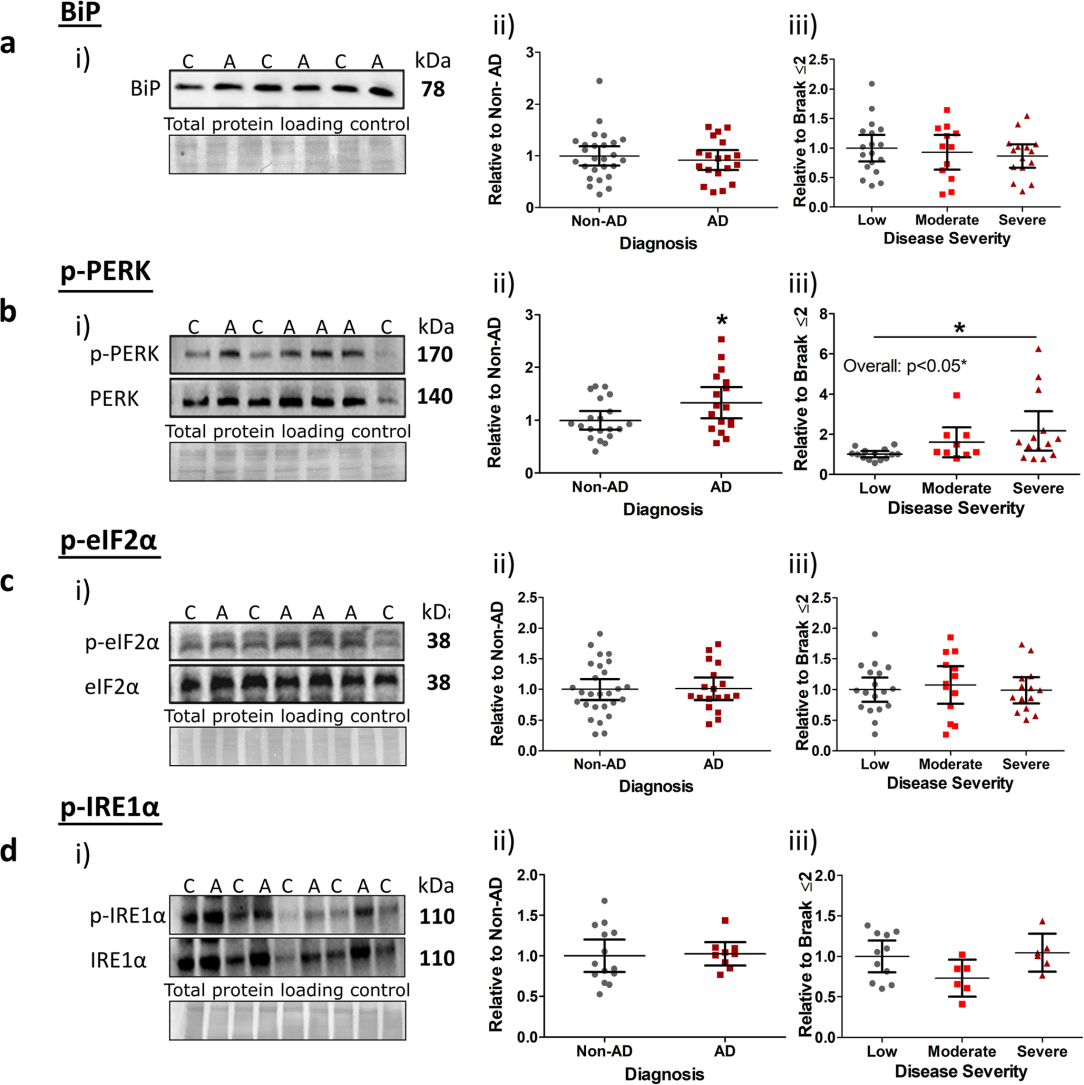
Selective elevation of UPR markers with end-stage AD pathology. i) Representative images of western blots probed for **a** BiP, **b** p-PERK/PERK, **c** p-eIF2α/eIF2α and **d** p-IRE1α/IRE1α. Molecular weights and diagnosis of non-AD (C) and AD (A) are indicated, alongside images of Coomassie total protein stain as loading controls. Markers were quantified according to ii) diagnosis and iii) disease severity (low, 0–2; moderate, Braak 3–4; severe, Braak 5–6). Significant post hoc inter-group differences are indicated (*). For all phosphorylated markers, scatter plots are shown as phospho-signal adjusted for total relative to appropriate controls. BiP (*n* = 45), p-PERK (*n* = 37), p-eIF2α (*n* = 45), p-IRE1α (*n* = 23) data displayed as scatter plots with means with 95% confidence intervals, *p < 0.05

To explore the proposed link between ER stress and AD, we next analysed levels of the ER chaperone protein, BiP, and UPR markers p-PERK, p-eIF2α and p-IRE1α. No changes in BiP were detected across AD cases compared to non-AD cases (Fig. 2a(i, ii)), or when analysed according to disease severity (Fig. 2a(iii)). Phosphorylated proteins (relative to total) yielded a significant increase for p-PERK in AD cases compared to non-AD controls (Fig. 2b(i, ii), *p* < 0.05), which could be attributed to an elevation between low and severe disease stages (Fig. 2b; Braak ≤2 vs. Braak 5–6, *p* < 0.05). Somewhat unexpectedly, this finding was not matched by p-eIF2α levels, a direct downstream target of p-PERK. Instead, phosphorylation levels of this protein remained unaffected for diagnosis status (Fig. 2c(i, ii)) and disease severity (Fig. 2c(iii)). Similarly, no changes in p-IRE1α expression emerged for any analysed parameter (Fig. 2d(i–iii)). Analysis of total PERK, eIF2α and IRE1α levels also reported no change (see Fig. S3). Taken together, these data show that only p-PERK is altered over the course of AD, and particularly in late AD stages, largely inconsistent with the coordinated activation of the UPR.

Analysis of UPR markers with cognitive scores and neuropathological staging found that only p-PERK, in line with our previous data, displayed any degree of correlation. This associated well with Braak staging (*p* < 0.05, *r* = 0.42, P.E. = ± 0.16) and CDR memory assessment (*p* < 0.05, *r* = 0.34, P.E. = ± 0.16), as well as yielding a strong trend with CERAD and CDR global scores (*p* = 0.061 and *p* = 0.059, respectively). Moreover, p-PERK correlated with phospho-tau markers. Interestingly, levels of total eIF2α displayed strong negative correlative trends with tau pathology (Table 3).

**Table 3:** Correlations of UPR markers with β-amyloid and tau pathology. Spearman’s rank correlations (*r*) of UPR markers with amyloid-β (Aβ) and tau pathology are illustrated. Negative correlations specified (↓). *p < 0.05, **p < 0.01, ^$^approaching significance. *N.S.* not significant

Marker	Soluble Aβ	Fibrillar Aβ	Oligomeric tau	Phosphorylated tau			Total tau
	MOAB	OC	TOC-1	PHF-1	AT-8	CP-13	HT-7
p-PERK	N.S.	N.S.	N.S.	^$^*p* = 0.053	*r = 0.37 ± 0.14	*r = 0.41 ± 0.14	N.S.
PERK	N.S.	N.S.	N.S.	N.S.	N.S.	N.S.	N.S.
p-eIF2α	N.S.	N.S.	N.S.	N.S.	N.S.	N.S.	N.S.
eIF2α	N.S.	N.S.	^$^*p* = 0.073 (↓)	^$^*p* = 0.06 (↓)	^$^*p* = 0.052 (↓)	N.S.	N.S.
p-IRE1α	N.S.	N.S.	N.S.	N.S.	N.S.	N.S.	N.S.
IRE1α	N.S.	N.S.	N.S.	N.S.	N.S.	N.S.	N.S.
BiP	N.S.	N.S.	^$^*p* = 0.072	N.S.	N.S.	N.S.	N.S.

### Neuro-inflammation

**Fig. 3:**
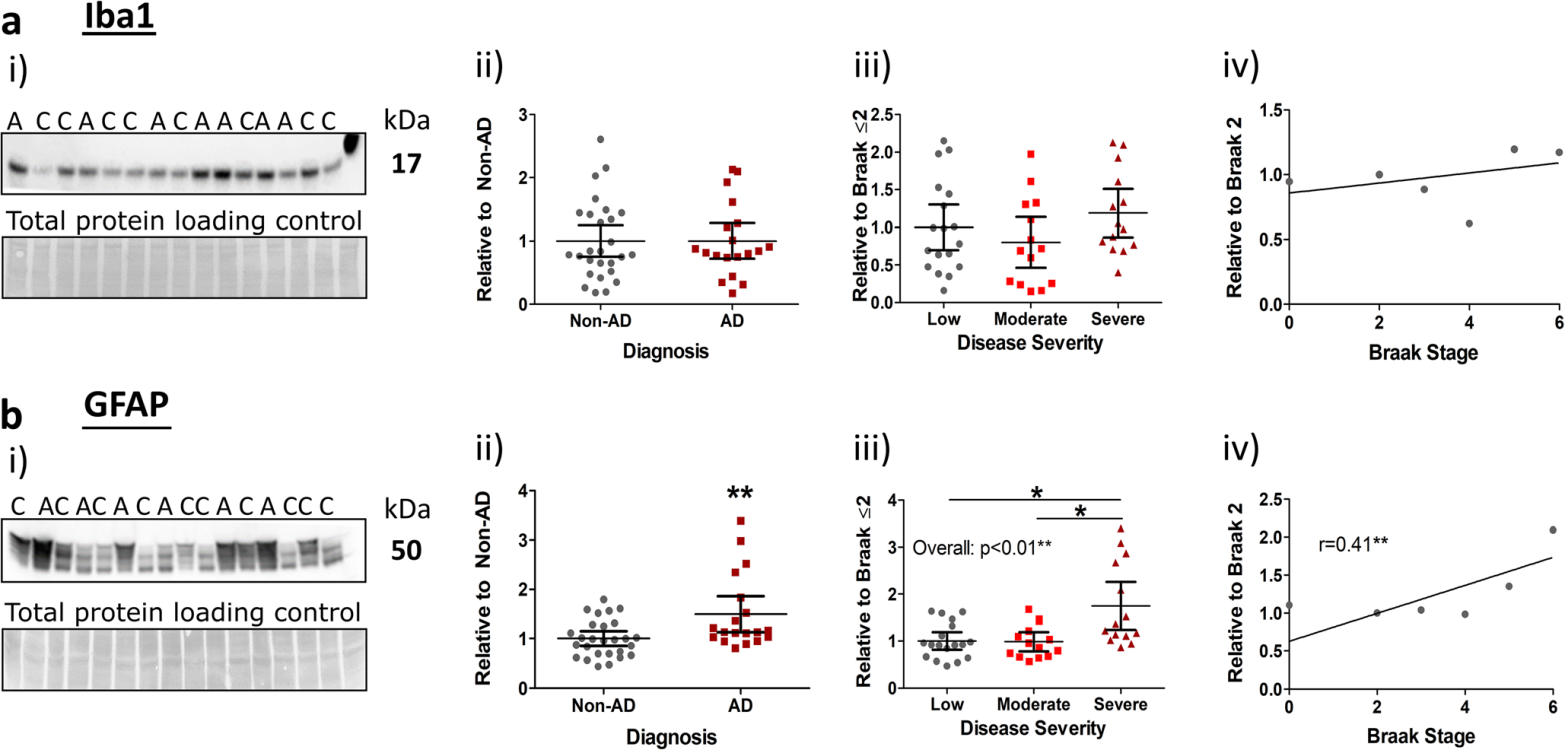
Astrocytic expression increases with end-stage AD pathology, but microglial expression remains unchanged. i) Example western blots stained for **a** Iba1 and **b** GFAP expression with molecular weights and individual diagnosis (non-AD (C), AD (A)) illustrated. Coomassie total protein loading controls are also shown. Stratified quantification of markers according to ii) diagnosis, iii) severity (low, Braak 0–2; moderate, Braak 3–4; severe, Braak 5–6) and individual Braak stages for correlation analysis (Spearman’s rank correlation (*r*)). Significant post hoc inter-group differences are indicated (*). Data for Iba1 (*n* = 46), GFAP (*n* = 46) given as scatter plots with mean values with 95% confidence intervals, *p < 0.05 and **p < 0.01

Neuro-inflammation, associated with activated astrocytes and microglia, was next probed alongside AD criteria. Surprisingly, we found no difference in the expression of the microglial marker, Iba1, across all analysed parameters (Fig. 3a(i–iv)). Conversely, the astrocytic marker, GFAP, demonstrated a significant elevation in accordance with AD diagnosis (Fig. 3b(i, ii), *p* < 0.01), and AD pathological severity (Fig. 3b(iii), *p* < 0.01). We further established that this increase was primarily due to an upregulation in severe AD cases (Braak ≤ 2 vs. Braak 5–6, *p* < 0.05; Braak 3–4 vs. Braak 5–6, *p* < 0.05), suggesting that astrocytic upregulation is a late-stage event. Not unexpectedly, GFAP was subsequently found to correlate well with Braak (neurofibrillary tangle) staging (Fig. 3b(iv); *r* = 0.41, *p* < 0.01).

In line with these findings, correlation analysis revealed that Iba1 did not associate with any measures of cognitive decline or hallmark AD pathology (all *p* > 0.05). GFAP, on the other hand, yielded weak to moderate correlations for neuropathological staging (CERAD, *r* = 0.44, P.E. = ± 0.14, *p* < 0.05) and measures of cognitive assessment (MMSE (*r* = − 0.39, P.E. = ± 0.15), CDR SOB (*r* = 0.43, P.E. = ± 0.14), *p* < 0.05; CDR global (*r* = 0.4, P.E. = ±0.14) and memory (*r* = 0.46, P.E. = ± 0.14), *p* < 0.01). Additionally, GFAP correlated well with both Aβ and tau measures (Table 4).

**Table 4:** Correlations of inflammatory markers with β-amyloid and tau pathology. Spearman’s rank correlations (*r*) of amyloid-β (Aβ) and tau pathology with Iba1 and GFAP are illustrated. *p < 0.05, **p < 0.01, **p < 0.001, ^$^approaching significance. *N.S.* not significant

Marker	Soluble Aβ	Fibrillar Aβ	Oligomeric tau	Phosphorylated tau			Total tau
	MOAB	OC	TOC-1	PHF-1	AT-8	CP-13	HT-7
Iba1	N.S.	N.S.	N.S.	N.S.	N.S.	N.S.	N.S.
GFAP	^$^*p* = 0.062	***r = 0.51 ± 0.13	*r = 0.34 ± 0.15	**r = 0.38 ± 0.14	***r = 0.48 ± 0.14	*r = 0.35 ± 0.14	**r = 0.53 ± 0.13

**Fig. 4:**
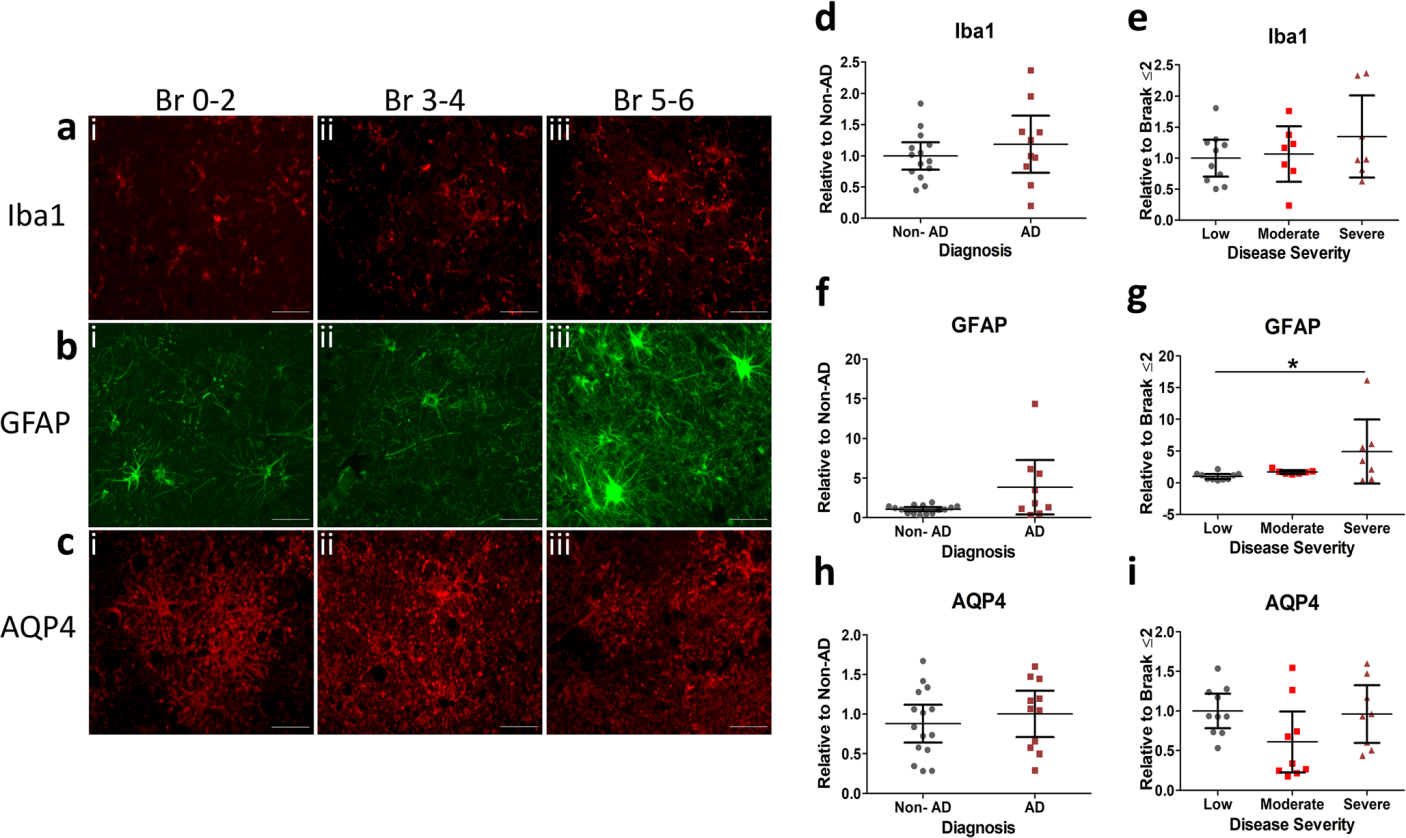
Immunohistochemical analysis confirmed end-stage upregulation of astrocytic expression. Representative images (magnification × 40, scale bar = 50 μm) of fixed brain sections of the lateral temporal cortex stained for **a** Iba1, **b** GFAP and **c** AQP4 with cases determined as i) Braak (Br) stage 0–2, ii) Br 3–4 and iii) Br 5–6 shown. Markers quantified as % area stained and stratified according to **d**, **f**, **h** diagnosis and **e**, **g**, **i** disease severity (low, Braak 0–2; moderate, Braak 3–4; severe, Braak 5–6). Significant post hoc inter-group differences are indicated (*). Quantification of Iba1 (*n* = 24), GFAP (*n* = 24), AQP4 (*n* = 26), reported as scatter plots with means with 95% confidence intervals, *p < 0.05

To further explore whether neuro-inflammation and specifically astrocyte activation emerge in later stages of the disease process, we carried out additional quantitative immunohistochemical analysis on a subset of AD cases (see Table S1 for cases used). Again, Iba1 immunostaining levels remained consistent across all Braak stages analysed (Fig. 4a) and reactivity was found unchanged in both diagnosed AD cases (Fig. 4d) and across disease severity (Fig. 4e). In contrast, GFAP staining across all Braak stages indicated an associated rise in astrogliosis. Little staining was detected in Braak 0–2, increasing in Braak 3–4 cases, and abundant in late stages 5–6 (Fig. 4b). Accordingly, GFAP levels were significantly increased across disease severity, particularly in severe AD cases (Fig. 4g). GFAP levels were not consistently upregulated across diagnosis status (Fig. 4f); however, this is likely due to higher variability within the AD group and the reduced number of respective cases analysed (*n* = 9). We also examined expression levels of the water channel AQP4, located on astrocytic end feet, which has been implicated in AD pathology due to its role in Aβ clearance [38]. No differences in AQP4 staining was seen across any Braak stages (Fig. 4c) and levels remained unchanged in diagnosed AD cases (Fig. 4h) and across severity (Fig. 4i). The unalterated status of AQP4 as part of AD pathology in the lateral temporal cortex was further supported by native state dot-blot measurements in a subset of cases, in which immunoreactivity was comparable between groups in all analytical parameters (see Fig. S4).

### Impact of Cohort Stratification and Co-variants

Given the potential influence of cohort stratification to alter the above statistical outcomes, the entire data set presented here was reanalysed following alternative stratification according to the NIA-AA guidelines reflecting the degree of AD-related neuropathological change. Consistently, analysis with non-parametric Kruskal-Wallis tests as well as Spearman’s rank correlations demonstrated largely the same outcome when classified under NIA-AA guidelines as to when grouped according to Braak stages (see Table S3). Aside from supportive correlations of p-PERK (*p* < 0.05, *r* = 0.39, P.E. = ± 0.16) and GFAP (*p* < 0.05, *r* = 0.35, P.E. = 0.15) with Thal phase, no further significant effect nor correlation with, Thal phase, APOE allele status or PART classification was reported for any marker analysed here. Similarly, no correlation was found with age or PMI for any measure.

## Discussion

This study sought to build on a previously well-characterised AD cohort [2, 3], quantifying and contextualising synaptic loss, ER stress and neuro-inflammatory pathology in the temporal lobe of post-mortem human AD tissue. Data were examined across neuropathological and cognitive scores and revealed that key candidates emerged late in the temporal cortex, more robustly associated with tau rather than Aβ pathology, and selectively correlated with cognitive decline.

### Synaptic Pathology

Synaptic dysfunction and loss is a defining feature of AD and is recognised as the best correlate of cognitive impairment [17, 41]. In line with previous reports [34, 42, 43], this study demonstrates a significant decrease in the expression of the postsynaptic protein, PSD-95, in AD cases. Interestingly, we observed no reduction in levels of the presynaptic marker, synaptophysin [43–45] or the reported early decrease in synaptic proteins, typically observed within the hippocampus or frontal cortex [16, 18, 37]. We must note that our employed method of investigating total expression levels is not a direct measure of functional synapses. Critically, however, we suggest that the differential outcome may largely depend on (1) The region investigated and (2) the composition of the study cohort.

Indeed, synaptic loss within the hippocampus [18, 19, 46] and the frontal cortex [17, 41, 47, 48] are frequently reported as early and correlative with cognitive decline. Yet, several post-mortem studies have highlighted a disconnect between these early affected regions and those observed elsewhere. For instance, the earliest synaptic loss may be confined to the hippocampus [19], and while frontal cortical synaptic loss may reflect global cognitive impairment, measures in the same cases revealed that superior temporal cortical loss may not [41]. Frontal lobe synaptic loss does also not correlate with decrease in the temporal lobes (reviewed in [49]). In line with our present findings, studies investigating the temporal lobe also reported synaptic decline in the more advanced pathology stages for most markers [44]. Particularly within the temporal lobes, postsynaptic dysfunction may indeed be more prominent, with reports of a post-synaptic drebrin decline earlier [44] or in the absence [50] of a synaptophysin decrease.

Somewhat independent of region, we note that studies which have clearly defined, low Braak stage (0–1) controls [37, 42], or those which limited their cohort to the extremes of pathology, more readily report robust synaptic loss [45]. This is in contrast to here and elsewhere [44], where a broader range of cases were analysed, mostly corroborating a later change in synaptic integrity. Such dependence on analytical strategy is typified by Mukaetova-Ladinska et al. [51] where statistically relevant differences were observed dependent on cohort grouping, ultimately leading the authors to report synaptic loss as a late occurrence. Interestingly, when analysed across individual Braak stages, an initial *increase* in synaptic markers was also reported, prior to a later decline, akin to the subtle increase in both PSD-95 and synaptophysin observed here within moderate cases. Such an initial upregulation of synaptic markers may be indicative of synaptic compensatory mechanisms, as suggested by others [52–55].

In the present cohort, modest associations with tau pathology but no correlation with Aβ pathology or CERAD neuropathological assessment were seen with PSD-95 levels. It must, however, be noted that the potential biphasic response in synaptic markers likely weakens such linear correlations. Nevertheless, the lack of association seen with Aβ is at odds with synaptotoxicity studies of this peptide [4, 56, 57], especially those which demonstrate a loss of PSD-95 following the local application of Aβ oligomers [58]. The synaptotoxicity of tau is less studied, yet, knock-in or overexpression of FTLD mutant human tau in mice is reported to reduce synaptic transmission and efficacy [59, 60] and several studies have indicated that Aβ-mediated synaptic impairment is in fact dependent on phospho-tau [61, 62].

Perhaps the most striking was the finding that neither synaptic marker correlated with any measures of cognitive decline. However, with the aforementioned disruptive nature of a biphasic synaptic response to linear correlation, and possible compensatory upregulation, it is plausible that the predictive value of cognitive decline associated with regional synaptic loss may not be homogenous throughout the brain [41].

It is interesting to note that removal of cases noted to have additional non-AD-related pathologies from our data set yielded different statistical outcomes, potentially suggesting that at least in the lateral temporal lobe, synaptic number and/or composition may be commonly affected by a variety of stressors. The individual differences with the cohort affected by co-morbidities may have masked a stronger contribution of Aβ and tau to synaptic dysfunction and its consequential impact on cognition. This reaffirms the necessity of detailed record keeping and considerations of comorbid confounders for quantitative pathological measures.

Collectively, current data suggest that robust synaptic loss in the lateral temporal lobe is a later-occurring event in the pathology of AD. Within this region, AD-driven synaptic dysregulation preferentially affects the postsynaptic compartment and is closely associated with the accumulation of pathological tau over Aβ species.

### ER Stress

We here also detected activation of the UPR in AD cases, although this appeared to be selective for p-PERK levels, which correlated with Braak stages, NIA-AA classifications, Thal phase and some measures of cognitive decline. The elevation of p-PERK was without a corresponding increase in p-eIF2α, the downstream substrate. Such observations are in line with the study of Bruch and colleagues where an increased p-PERK was reported alongside suppressed total eIF2a levels [63].

Previous studies in AD cases have observed increased UPR markers, e.g. pre-tangle phospho-tau bearing neurons of the hippocampus expressed elevated levels of p-PERK and p-eIF2α [20, 22, 26]. Similar findings have also been reported in several tauopathy variants [27]. More recent work by Duran-Aniotz and colleagues reported an elevation of p-IRE1α in the hippocampus of AD cases, in close association with Braak staging [21]. This close association with pre-tangle pathology has led to the notion that the UPR is activated early in the course of the disease and may contribute to disease pathogenesis. Here, associations of p-PERK with pathological tau markers were also established, but elevated levels were confined to late-stage pathological cases and occurred without the corroborative changes in other key markers. The delayed activation of the UPR over the course of AD within the neocortical regions is further supported by similar findings within the frontal cortex, in which p-PERK was selectively elevated in Braak stage 6 cases, in the absence of enhanced p-eIF2α levels [64]. Equally, quantification of p-PERK immunostaining in AD cases has revealed a more modest activation within the frontal cortex as compared to the hippocampus within the same cases [22]. Nevertheless, certain cortical areas may be more susceptible to ER stress than others, given the regional specificity in which BiP is found elevated [65].

It must also be considered that many of the studies reporting early, multi-marker activation of the UPR did so through immunohistological detection [20–22, 26], in contrast to immunoblot-based studies (here and, e.g. [64]), which typically report later, incomplete activation. Only some sub-populations of neurons within each region are likely to experience a degree of ER stress sufficient to activate the UPR and, consequently, more global assessments via immunoblot may mask selective differences. Thus, immunoblot techniques are able to establish changes in severe stages of disease when pathology is substantial, while immunohistochemical techniques likely provide a better resolution regarding cell type- and region-specific changes.

Independent of the method of detection, and consistent with our findings, post-mortem studies have invariably found a close association of the UPR with tau pathology [20–22, 26]. Such a specific association is surprising given that in addition to tau [24], both Aβ oligomers [66] and BACE1 products [67] have been demonstrated to induce the UPR. Furthermore, in AD models, the UPR is linked to the promotion of Aβ production via enhanced BACE1 expression [25, 68] and diminished APP turnover [21]. Nevertheless, here we found no evidence of enhanced p-eIF2α levels and likewise, we are unable to support the association of reduced protein synthesis, via p-eIF2α, with synaptic failure and memory deficits which has been reported in animal models [23, 28, 29, 63]. As for all human tissue studies, the impact of post-mortem interval (PMI) on phosphorylated protein levels must be considered [69, 70].

Given that in this cohort we have previously observed significant tau and Aβ pathology within intermediate Braak stages [2, 3], our data illustrate that specific UPR markers track disease pathology late in the disease course and, thus, may be reactive to pathology in the temporal lobe rather than facilitatory of pathological spread. Nevertheless, UPR activation may further exacerbate degenerative disease processes.

### Neuro-inflammation

Similar to synaptic loss, neuro-inflammation is a characteristic feature of AD. In line with this and previous findings, we confirmed a significant upregulation of GFAP in AD cases compared to controls [34, 35, 71], particularly in severe (Braak stage 5–6) AD cases [71]. We extended on these prior reports, establishing the robust upregulation of GFAP within the AD temporal lobe as restricted to late-stage AD cases. This is somewhat in contrast to reports of a biphasic response in MCI and AD patients as measured by the monoamine oxidase B inhibitor PET ligand (^11^C-deuterium-l-deprenyl; DED), which is supportive of an early upregulation and later decline of astrogliosis [38, 72]. However, such reports have indicated that these early changes are region specific, observing alterations in DED retention times only in the frontal and parietal cortex [38]. Furthermore, a disconnect between DED retention and GFAP staining in an FAD mouse model has raised concerns over the specificity of the ligand [73], suggesting that at present, there is no clear evidence for an early inflammatory (astroglia) reaction in human AD [74]. Nevertheless, several cross-sectional AD animal model studies found an early upregulation of astrocytes, preceding Aβ plaque deposition and correlative with soluble Aβ [73, 75, 76].

Here, we observed GFAP correlations with neuropathological Braak stage, CERAD, NIA-AA and Thal phase assessment scores as well as phospho-tau and Aβ. This may be associated with a late-stage inflammation as a result of astrocyte accumulation around Aβ species [77], although others [78] focussing on the temporal cortex, also observed greater correlative strength between pathological tau vs. Aβ pathology. Given astrogliosis is evident in response to numerous neurodegenerative insults (reviewed by Ransohoff [79]), the principal driving force for their activation may largely vary depending on the extent of regional pathology.

Strikingly, we observed a modest but significant correlation between GFAP levels and all measures of cognitive decline. This was somewhat unexpected, considering the lack of a corresponding relationship with synaptic measures. Nevertheless, substantial evidence supports the underpinning of cognition via astrocytic facilitation of synaptic signalling and plasticity, and its subsequent disruption during pathology [80]. Accordingly, the inhibition of excessive astrocytic signalling may protect against cognitive decline, independent of Aβ, as shown in a FAD mouse model [81]. Collectively, out of all markers investigated, GFAP correlated best with both Aβ and tau pathology as well as cognitive deficits, suggesting that astrocytes track both disease progression and pathology. However, as elevations of GFAP emerged after tau and Aβ, astrogliosis appears to be reactive to initial pathology.

Despite the fact that GFAP and thus astrogliosis can be considered a robust indicator of the disease, we failed to observe any significant overt change in the water channel AQP4, thought to be involved in the glymphatic clearance of Aβ [82, 83] and proposed to be disrupted in AD [36]. Nevertheless, our measurements of AQP4 were either sampled at random within the temporal lobe via histology or represented total tissue levels in immunoblots and thus were not localised to the site of plaques, where greatest changes have been identified [84, 85]. We similarly detected no changes in the expression levels for Iba1, a general marker of microglia. Indeed, notwithstanding the well-documented activation of microglia in AD [15], a recent systematic review of microglial markers in post-mortem human AD tissue reported that half of the publications quantifying Iba1 levels detected no change in expression compared to controls. Interestingly, these heterogeneous results based on Iba1 labelling of all microglia were not seen with a marker specific to activated microglia, CD68, which was consistently elevated in AD cases [86]. In line with this, it has been proposed that increased activation as opposed to proliferation of microglia occurs in AD [86, 87]. As a result, future work will be required to evaluate the context of microglial responses in relation to AD pathologies.

### Concluding Remarks

In summary, our data suggest that specific synaptic, ER stress and neuro-inflammatory markers are affected in late AD in the temporal gyrus. This study does not rule out dysregulation earlier elsewhere in the brain or the differential effect on specific neuronal subtypes. However, given that our previous analysis of temporal cortical tau and Aβ pathology within the same cohort has demonstrated the co-localisation of soluble Aβ and tau pathology at intermediate stages of neuropathological severity (Braak NFT stages 2–3), the pathways studied here do not precede the spread of tau or Aβ pathology and are likely secondary events. Strong associations of affected measures were detected with phospho-tau species, and less so with Aβ pathology, thus being at odds with the assumption that the Aβ cascade primarily drives disease processes. Though we cannot rule out the contributions of ER stress and neuro-inflammation to the initial emergence of tau and Aβ pathology at the site of origin (e.g. EC/hippocampus for tau [6], orbitofrontal cortex and inferior temporal gyrus for Aβ [8, 9], or early synaptic loss within these regions), our data suggest that neither of these pathways precedes the pathology in the middle temporal gyrus. Sequential progression of pathology throughout the brain, distinct from pathogenesis, may be more dependent on the associated biochemical properties of protein seeding as suggested by a prion-like spread [88]. Nevertheless, the ensuing activation of detrimental degenerative cascades likely exacerbates the overall degeneration. Consequently, therapeutic targeting of the underlying mechanisms may not halt the disease, yet may serve to ameliorate and thus delay further cellular dysfunction throughout the disease course.

## Electronic Supplementary Material

ESM 1 (DOCX 2593 kb)

ESM 2 (PDF 341 kb)

## Data Availability

The datasets used and/or analysed during the current study are available from the corresponding authors on reasonable request.
