# Supplementary material for: Synaptic Loss, ER Stress and Neuro-Inflammation Emerge Late in the Lateral Temporal Cortex and Associate with Progressive Tau Pathology in Alzheimer’s Disease
Source: Mol Neurobiol. 2020 Jun 8;57(8):3258–72. doi: 10.1007/s12035-020-01950-1 (PMC7340653; doi:10.1007/s12035-020-01950-1)
Supplement: Supplementary file 1 — (DOCX 2593 kb) [file 12035_2020_1950_MOESM1_ESM.docx]

**SUPPLEMETARY INFORMATION**

**Title**

Synaptic Loss, ER Stress and Neuro-inflammation Emerge Late in the Lateral Temporal Cortex and Associate with Progressive Tau Pathology in Alzheimer’s disease

**Authors**

Heather Buchanan^1^, Murray Mackay^1^, Kerri Palmer^1^, Karolína Tothová^1^, Miroslava Katsur^1^

Bettina Platt^1^, David J. Koss^1, 2^

**Affiliation**

^1^School of Medicine, Medical Sciences and Nutrition, University of Aberdeen, Foresterhill, Aberdeen, AB25 2ZD, UK.

^2^ Institute of Neuroscience, Newcastle University, Campus for Ageing and Vitality, Newcastle upon Tyne, NE4 5PL, UK.

**Corresponding authors**

Dr. David J. Koss: [David.Koss@ncl.ac.uk](mailto:David.Koss@ncl.ac.uk) Tel: +44 (0) 191 208 1213

Prof Bettina Platt: [B.Platt@abdn.ac.uk](mailto:B.Platt@abdn.ac.uk) Tel: +44 (0)1224 437 402

| Bank | Case | Sex | Age | Braak Stage | CERAD | Thal Phase | NIAA -AA | APOƐ | PART | PMI | MMSE | CDR Global | CDR Mem | Analysis |
| --- | --- | --- | --- | --- | --- | --- | --- | --- | --- | --- | --- | --- | --- | --- |
| Lon | 1 | M | 74 | 0 | C0 | 0 | None | 3/3 | Non | 23 | 30 | 0 | 0 | WB/ IHC |
| Lon | 2 | M | 78 | 0 | C0 | 0 | None | 3/3 | Non | 11 | 26 | 0 | 1 | WB/ IHC |
| Bris | 3 | M | 78 | 0 | C0 | 0 | None | 3/4 | Non | 56 | 29 | 0 | 0.5 | WB/ IHC |
| Bris | 4 | M | 96 | 2 | C0 | 0 | None | 2/4 | Def | 21 | 26 | 0 | 0 | WB/ IHC |
| Oxf | 5 | F | 84 | 2 | C0 | 3 | Low | 3/3 | Non | 72 | 30 | 0 | 0 | WB/ IHC |
| Oxf | 6 | F | 84 | 2 | C0 | 4 | Low | 3/3 | Non | 30 | 29 | 0.5 | 1 | WB/ IHC |
| Oxf | 7^1^ | F | 79 | 2 | C0 | 1 | Low | 3/3 | Poss | 18 | 26 | 0 | 0 | WB/ IHC |
| Oxf | 8^2^ | M | 85 | 2 | C0 | 2 | Low | 2/4 | Poss | 24 | - | 2 | 0 | WB/ IHC |
| Oxf | 9 | F | 89 | 2 | C1 | 5 | Low | 2/3 | Non | 12 | - | - | - | WB/ IHC |
| Lon | 10 | F | 90 | 2 | C0 | 0 | None | 3/3 | Def | 44 | 27 | 0 | 0 | WB |
| Lon | 11^3^ | F | 90 | 2 | C0 | 0 | None | 3/3 | Def | 87 | 29 | 0 | 0 | WB |
| Bris | 12 | M | 78 | 2 | C0 | 0 | None | 3/3 | Def | 51.5 | 30 | 0 | 0 | WB/ IHC |
| Bris | 12 | F | 94 | 2 | C0 | 2 | Low | 3/4 | Poss | 29.5 | 28 | 0.5 | 0.5 | WB |
| Man | 14^4^ | F | 103 | 2 | C1 | 3 | Low | 3/3 | Non | 41 | 0 | 3 | 3 | WB |
| Man | 15 | M | 74 | 2 | C1 | 3 | Low | 3/3 | Non | 92 | 0 | 3 | 3 | WB |
| Man | 16 | F | 91 | 2 | C1 | 2 | Low | 3/3 | Poss | 78 | - | - | - | WB |
| New | 17 | M | 80 | 2 | C0 | 0 | None | 3/3 | Def | 16 | 29 | 0 | 0 | WB |
| New | 18 | M | 88 | 2 | C0 | 3 | Low | 3/3 | Non | 26 | 26 | 0 | 0 | WB |
| Lon | 19^5^ | F | 89 | 3 | C2 | 2 | Inter | 3/3 | Poss | 56 | 29 | 0 | 0.5 | WB/ IHC |
| Lon | 20 | F | 84 | 3 | C2 | 2 | Inter | 3/4 | Poss | 89 | 30 | 0 | 0 | WB |
| Lon | 21 | F | 87 | 3 | C0 | 0 | None | 2/3 | Def | 67 | - | - | - | WB/ IHC |
| Lon | 22 | M | 95 | 3 | C2 | 2 | Inter | 3/3 | Poss | 26 | 29 | 0 | 0 | WB/ IHC |
| Bris | 23 | F | 85 | 3 | C0 | 0 | None | 2/2 | Def | 13.5 | 29 | 0 | 0.5 | WB |
| Man | 24 | F | 86 | 3 | C2 | 4 | Inter | 3/4 | Non | 36 | 23 | 2 | 2 | WB/ IHC |
| Man | 25 | M | 78 | 3 | C2 | 5 | Inter | 3/4 | Non | 42 | 0 | 3 | 3 | WB |
| Man | 26 | F | 92 | 3 | C2 | 4 | Inter | 3/3 | Non | 101 | 0 | 3 | 3 | WB |
| New | 27 | M | 92 | 3 | C0 | 1 | Low | 3/4 | Poss | 50 | 20 | 1 | 1 | WB |
| Lon | 28 | F | 88 | 4 | - | - | - | 3/3 | - | 33 | 4 | 1 | 3 | WB/ IHC |
| Lon | 29 | M | 80 | 4 | C2 | 3 | Inter | 3/4 | Non | 51 | 8 | 2 | 2 | WB/ IHC |
| Oxf | 30 | F | 88 | 4 | C2 | 5 | Inter | 3/4 | Non | 44 | 20 | 2 | 2 | WB/ IHC |
| Man | 31 | M | 77 | 4 | C2 | - | - | 4/4 | - | 87 | 0 | 3 | 3 | WB |
| Man | 32 | F | 81 | 4 | C3 | 5 | Inter | 3/3 | Non | 26 | - | - | 3 | WB |
| Oxf | 33 | M | 85 | 5 | C3 | 5 | High | 3/4 | Non | 24 | 5 | 3 | 3 | WB/ IHC |
| Oxf | 34 | M | 82 | 5 | C3 | 5 | High | 3/4 | Non | 72 | 0 | 3 | 3 | WB/ IHC |
| Lon | 35 | M | 88 | 5 | C1 | - | - | 3/4 | Non | 66 | 23 | 1 | 1 | WB |
| Lon | 36 | M | 0 | 5 | C3 | - | - | 3/3 | Non | 78 | 0 | 3 | 3 | WB |
| Bris | 37 | F | 82 | 5 | C3 | 4 | High | 3.4 | Non | 22 | 0 | 3 | 3 | WB/ IHC |
| New | 38 | M | 82 | 5 | C3 | 5 | High | 3/4 | Non | 41 | 0 | 3 | 3 | WB |
| Lon | 39 | M | 79 | 6 | C3 | 2 | Inter | 3/4 | Non | 24 | 0 | 3 | 3 | WB/ IHC |
| Lon | 40 | M | 71 | 6 | C3 | 2 | Inter | 3/4 | Non | 20 | 0 | 3 | 3 | WB/ IHC |
| Lon | 41 | M | 79 | 6 | C3 | 3 | Inter | 3/4 | Non | 38 | 0 | 3 | 3 | WB/ IHC |
| Lon | 42 | M | 90 | 6 | C3 | 2 | Inter | 3/4 | Non | 69 | 0 | 3 | 3 | WB/ IHC |
| Bris | 43 | F | 89 | 6 | C3 | 4 | High | 3/4 | Non | 22 | 0 | 2 | 2 | WB/ IHC |
| New | 44 | F | 87 | 6 | C3 | 5 | High | 3/4 | Non | 51 | 0 | 3 | 3 | WB |
| New | 45 | F | 89 | 6 | C3 | 4 | High | 3/4 | Non | 65 | 7 | 2 | 2 | WB |
| New | 46 | F | 84 | 6 | C3 | 5 | High | 3/4 | Non | 52 | 5 | 3 | 3 | WB |
| Man | 47 | M | 87 | 4 | C2 | 4 | High | 4/4 | Non | 58 | - | - | - | IHC |
| Man | 48 | M | 77 | 3-4 | C3 | - | - | 3/4 | Non | 162 | - | - | - | IHC |
| Man | 49 | M | 88 | 3-4 | C2 | 2 | Inter | 3/4 | Non | 114 | - | - | - | IHC |

**Table S1** Biographical information of study cohort. Individual cases (indicated by case number) are shown alongside corresponding sex and age. Neuropathological scores of Braak stage, CERAD score, Thal phases, National Institute on Aging and Alzheimer’s Association (NIA-AA; guidelines reporting None, Low, Intermediate (Inter) or High neuropathic change related to AD , Primary Age related Tauopathy (PART) neuropathological criteria reporting if cases as Non, Possible (Poss) or Definite (Def) PART are also reported. Additionally, apolipoprotein Ɛ4 (APOƐ4) allele status, post mortem-interval (PMI), alongside cognitive scores mini-mental state examination (MMSE) score and clinical dementia rating (CDR) global and memory score are shown. The brain banks (London (Lon), Bristol (Bris), Manchester (Man), Oxford (Oxf) and Newcastle (New)) from which they were sourced and the type of analysis (western blot (WB) or immunohistochemistry (IHC)) conducted on each case is also indicated. - = information not available. Samples excluded based on additional pathologies are underlined and were omitted due to: ^1^microscopic foci of metastatic tumour, ^2^brainstem haemorrhage, ^3^TDP-43 pathology, ^4^TDP-43 pathology, ^5^neuroendocrine carcinoma.

| **Antibody** | **Epitope** | **Dilution** | **Supplier** |
| --- | --- | --- | --- |
| PSD-95 | aa 50-150 | 1:1000 | Abcam |
| Synaptophysin | aa 250- 350 | 1:10000 | Abcam |
| IBA-1 | C- terminus | 1:1000 | Wako |
| GFAP | Not reported | 1:1000 | Sigma |
| IRE1α | Not reported | 1:1000 | Cell signalling |
| p-IRE1α | p- Ser724 | 1:1000 | ThermoFisher |
| eIF2α | C- terminus | 1:1000 | Cell Signalling |
| p-eIF2α | p- Ser51 | 1:1000 | Cell Signalling |
| PERK | Not reported | 1:1000 | Cell Signalling |
| p-PERK | p- Thr960 | 1:1000 | Cell Signalling |
| BiP | aa 550- 650 | 1:1000 | ABclonal |

**Table S2** Antibodies used for western Blots. Those specific for phosphorylated proteins are listed as p-. Epitope residue (Serine (ser), Threonine (Thr)) and amino acid (aa) sequence is also given.

| NIA-AA | | |
| --- | --- | --- |
| Markers | Kruskal-Wallis | Spearman’s correlation |
| Synaptic | | |
| PSD-95 | Overall=**  Post-hoc;  None cf. Inter=*  None cf. High=* | N.S. |
| Synaptophysin | N.S. | N.S. |
| ER-Stress | | |
| BiP | N.S. | N.S. |
| p-PERK | Overall=**  Post-hoc;  None cf. High=*  Low cf. High=* Inter cf. High=* | **, r=0.5, P.E=±0.13 |
| Total-PERK | N.S. | N.S. |
| p-eIF2α | N.S. | N.S. |
| Total-eIF2α | N.S. | N.S. |
| p-IRE1α | N.S. | N.S. |
| Total-IRE1α | N.S. | N.S. |
| Neuroinflammation | | |
| GFAP (WB) | Overall=**  Post-hoc;  Low cf. High=* | **, r=0.4, P.E=±0.14 |
| IBA-1 (WB) | N.S. | N.S. |
| GFAP (IHC) | Overall=**  Post-hoc;  None cf. High=* | **, r=0.58, P.E=±0.18 |
| IBA-1 (IHC) | N.S. | N.S. |
| APQ4 (IHC) | N.S. | N.S. |

**Table S3** Analytical outcomes of data following National Institute on Aging and Alzheimer’s Association (NIA-AA) guidelines reporting None, Low, Intermediate (Inter) or High neuropathic changes related to AD. Results of non-parametric Kruskal-Wallis, Dunn’s post-hoc analysis and spearman’s correlation (r) with calculated probable error of coefficient. For neuroinflammation, western blot (WB) or immunohistochemistry (IHC) is indicated. *= p < 0.05, **=p < 0.01 and N.S. = not significant.


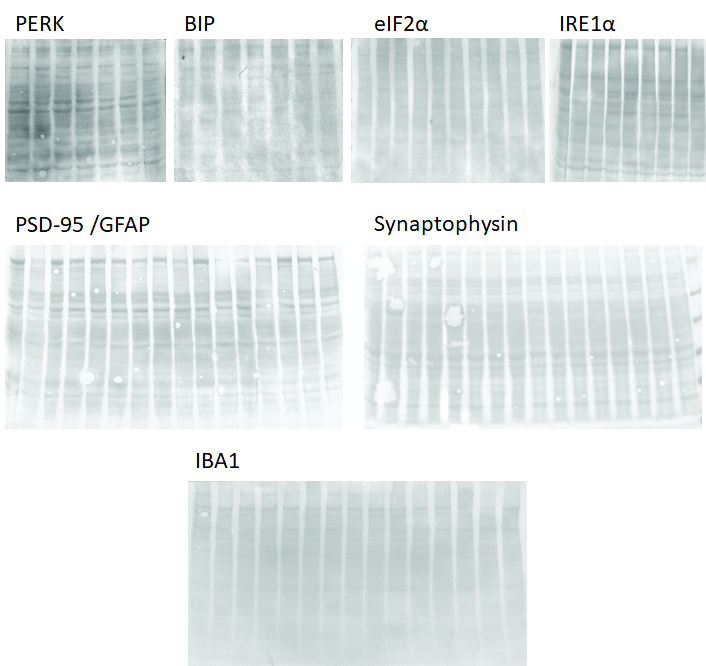


**Fig. S1** Example images of Coomassie total protein stained nitrocellulose membranes, relating to examples of ECL images of western blots provided in main manuscript. Densitometric measurements were taken from each lane for normalisation of ECL measures specific to target protein. Note that some membranes were used for the detection of more than one antigen (PSD-95 and GFAP)


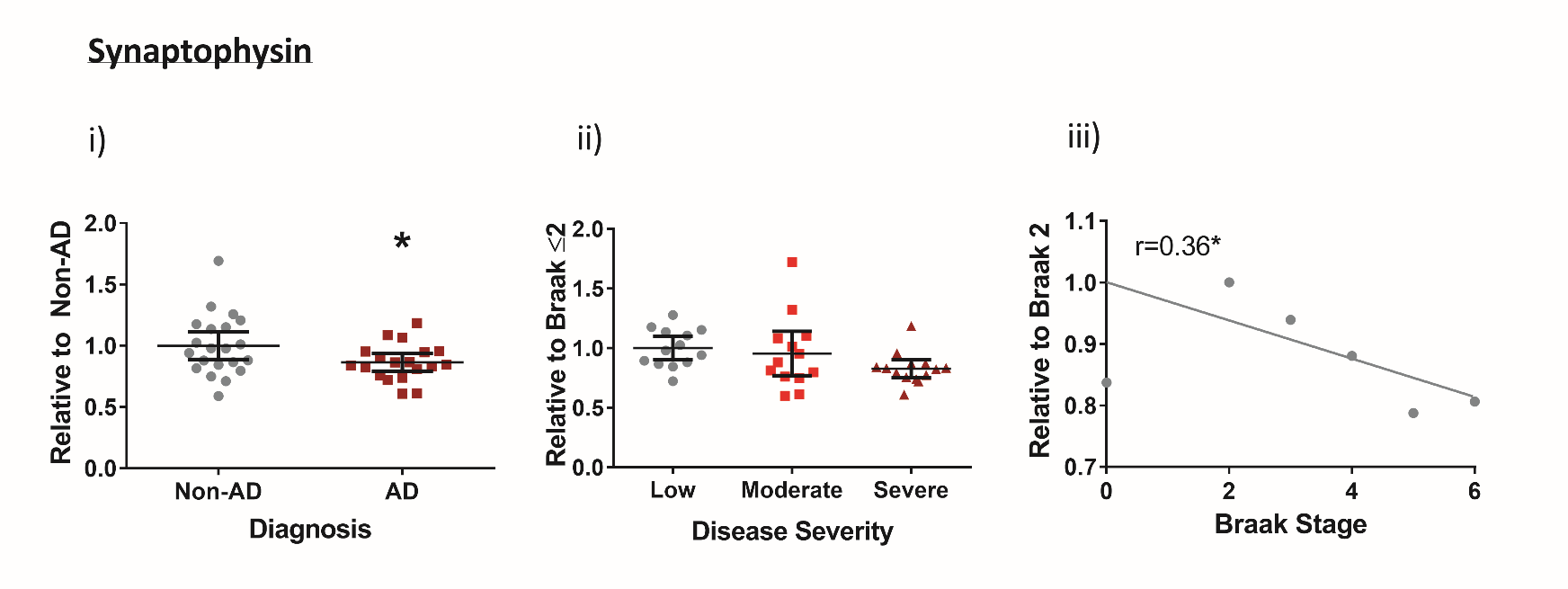


**Fig. S2** Exclusion of samples with overt secondary pathologies reveals a decrease in synaptophysin in AD. Quantified markers were analysed according to i) diagnosis, ii) disease severity (low, Braak 0-2; moderate, Braak 3-4; severe, Braak 5-6) and iii) correlated (Spearman’s r analysis) with individual Braak stage. Synaptophysin data (N = 46) provided as scatter plots and means with 95% confidence intervals. * = p < 0.05.


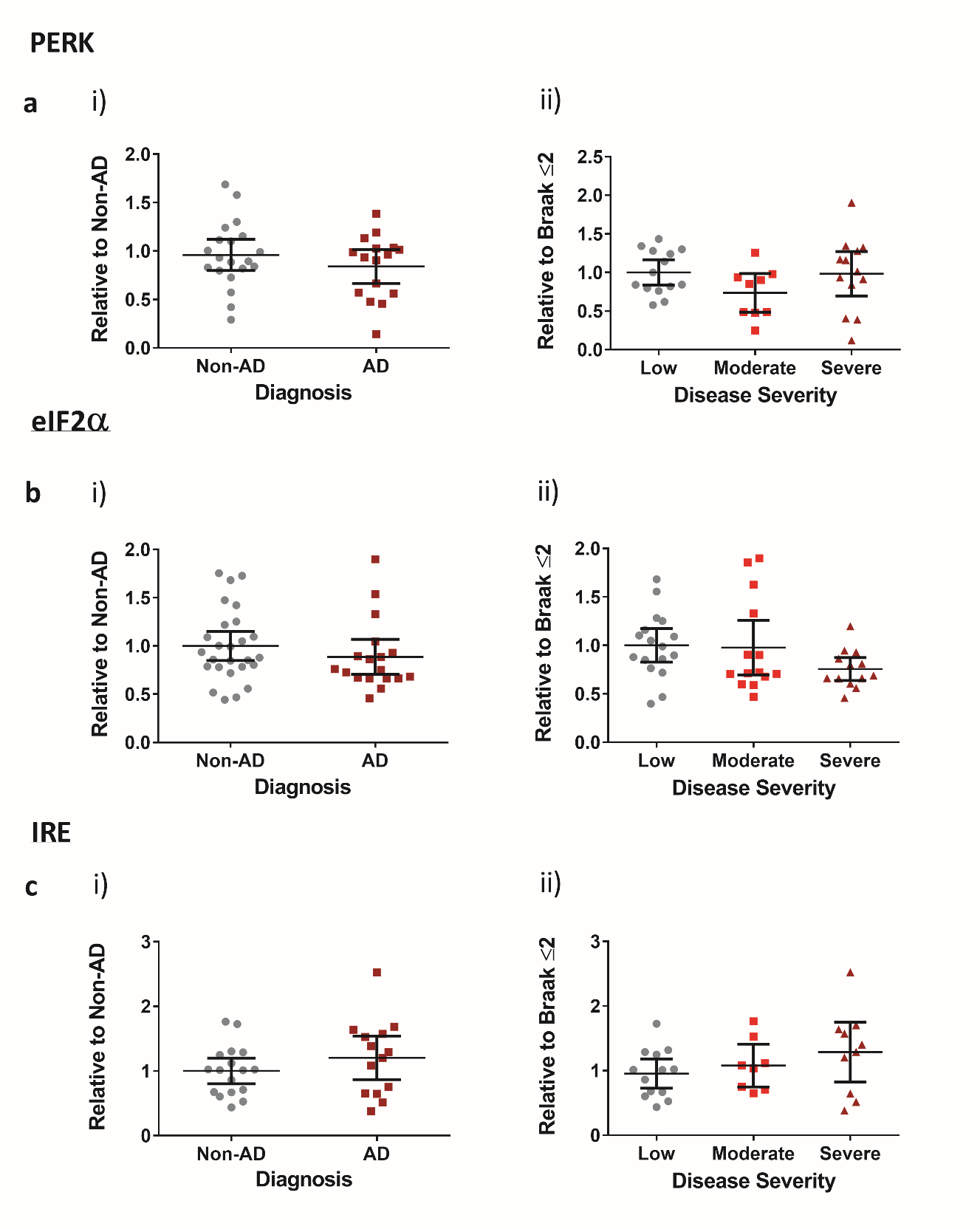


**Fig. S3** Total protein levels of UPR markers are unchanged in AD. Markers were quantified according to i) diagnosis and ii) disease severity (low, Braak 0-2; moderate, Braak 3-4; severe, Braak 5-6) for (a) PERK, (b) eIF2α and (c) IRE. PERK (n = 37), eIF2α (n = 45) and IRE (n = 23) data displayed as scatter plots with means with 95% confidence intervals.


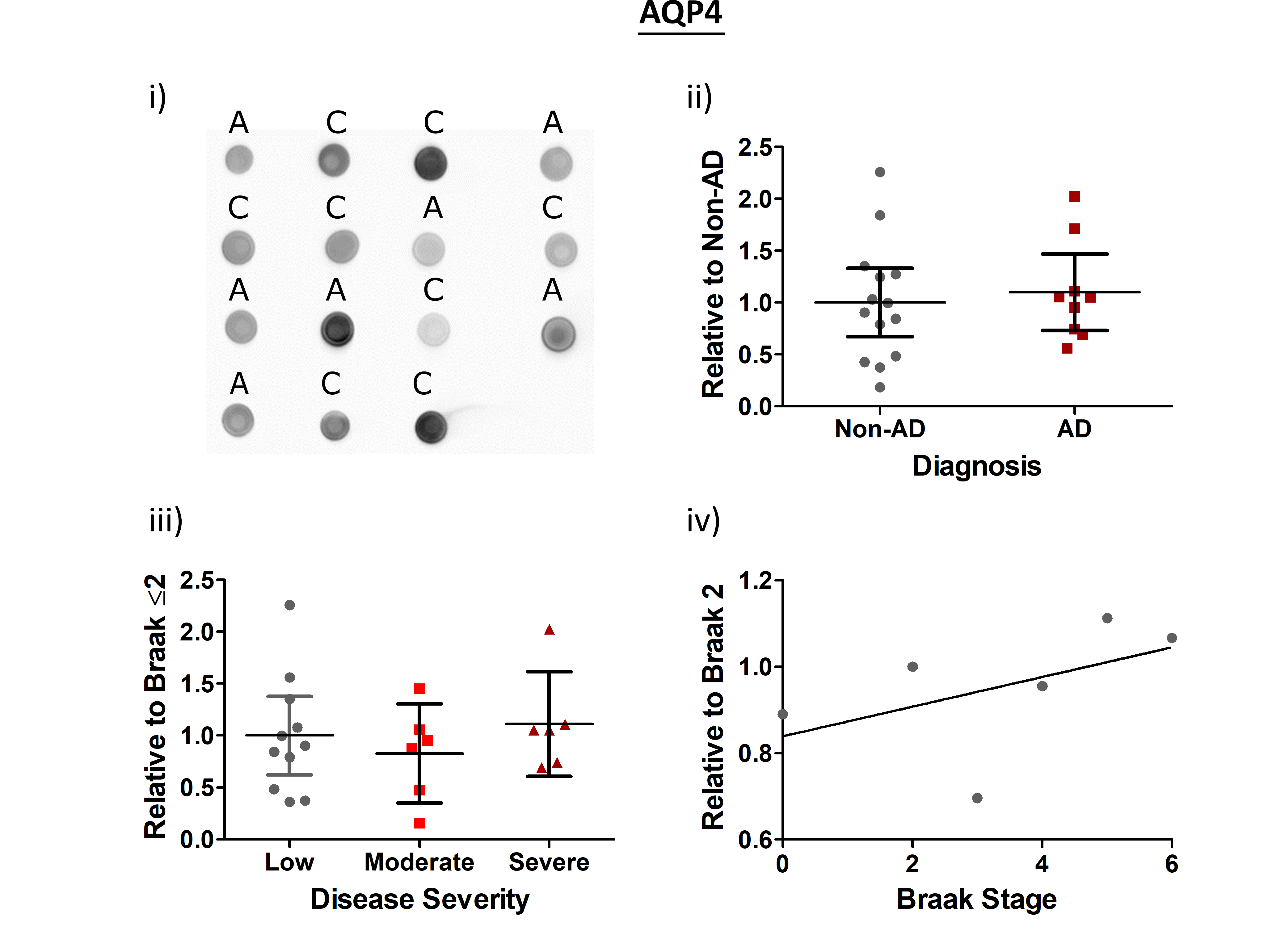


**Fig. S4** AQP4 levels are unchanged in AD. i) Example dot blots probed for AQP4 with individual diagnosis illustrates (non-AD (C), AD (A)). Quantified according to ii) diagnosis, iii) disease severity (low, Braak 0-2; moderate, Braak 3-4; severe, Braak 5-6) and iv) individual Braak stages for correlation analysis (Spearman’s r correlation). Data for AQP4 (n= 23) given as scatter plots with means and 95% confidence intervals.
