# Supplementary material for: Synaptic Loss, ER Stress and Neuro-Inflammation Emerge Late in the Lateral Temporal Cortex and Associate with Progressive Tau Pathology in Alzheimer’s Disease
Source: Mol Neurobiol. 2020 Jun 8;57(8):3258–72. doi: 10.1007/s12035-020-01950-1 (PMC7340653; doi:10.1007/s12035-020-01950-1)
Supplement: Supplementary file 2 — (PDF 341 kb) [file 12035_2020_1950_MOESM2_ESM.pdf]

Batch comparison –western blots
PSD-95 Batch: p>0.05

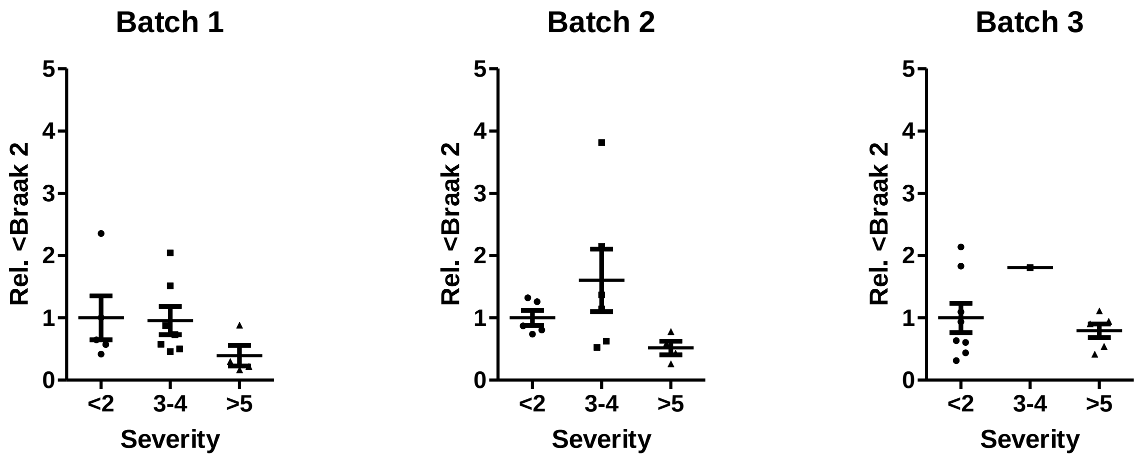

Mean of Braak >5 is lower in all batches compared to Braak<2 group

Perk Batch: p>0.05

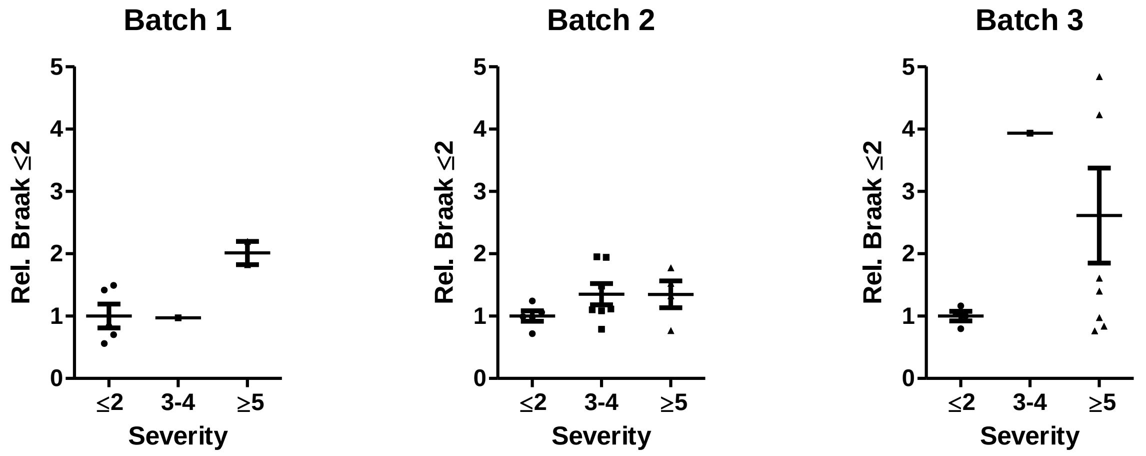

Mean of Braak >5 is higher in all batches compared to Braak<2 group

Batch comparison –Immunohistochemistry
GFAP Batch: p>0.05

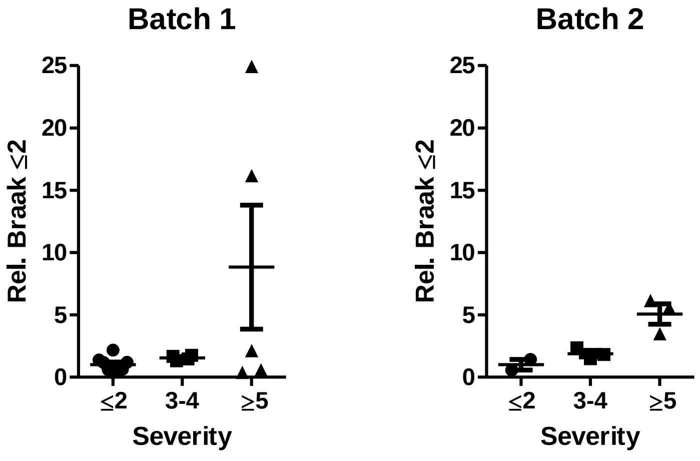

Mean of Braak >5 is higher in all batches compared to Braak<2 group
